# Severe Hypoglycemia-Induced Seizure in a Four-Year-Old Following Initiation of Nadolol for Supraventricular Tachycardia

**DOI:** 10.7759/cureus.103387

**Published:** 2026-02-10

**Authors:** Zain M Al Muqbel, Ali M Haider Ali, Rami Al Ansari, Nasser Mansoor, Abdulrahman Al-Majmuei

**Affiliations:** 1 Accident and Emergency, Salmaniya Medical Complex, Muharaq, BHR; 2 Medicine, Royal College of Surgeons in Ireland - Bahrain, Muharaq, BHR

**Keywords:** beta blocker adverse effects, hypoglycemia, nadolol, pediatric seizure, supraventricular tachycardia (svt)

## Abstract

Severe hypoglycemia is a rare but potentially life-threatening complication of non-selective beta-blockers in children. We report a four-year-old boy who developed a prolonged generalized tonic-clonic seizure shortly after initiation of nadolol for supraventricular tachycardia. The bedside random blood glucose was 0.9 mmol/L. He was promptly treated with intramuscular glucagon and intravenous dextrose, resulting in stabilization. History included refractory supraventricular tachycardia previously managed with esmolol and propranolol. Nadolol had been started the evening prior to the seizure. Laboratory evaluation showed low C-peptide with otherwise normal fasting glucose. Echocardiography revealed mild mitral regurgitation with preserved systolic function. Nadolol was discontinued, and propranolol was carefully titrated under continuous cardiac and glucose monitoring. Endocrinology recommended a formal fasting study prior to discharge. Clinicians should monitor glucose closely and educate caregivers on early recognition of hypoglycemia when initiating non-selective beta-blockers in young children.

## Introduction

Beta-blockers remain a cornerstone in pediatric arrhythmia management, including supraventricular tachycardia. Non-selective agents such as nadolol can impair glycogenolysis and gluconeogenesis, increasing the risk of hypoglycemia. Furthermore, beta-blockers may mask adrenergic warning signs such as tremor, palpitations, or anxiety, delaying recognition. Early identification and reporting of such adverse events are crucial to guide safe prescribing practices and enhance clinician awareness in vulnerable pediatric populations [1-3]. Severe hypoglycemia associated with beta-blocker therapy in children is considered rare, with most available evidence derived from pharmacovigilance studies and isolated case reports rather than large prospective studies [1]. 

## Case presentation

A four-year-old boy experienced a generalized tonic-clonic seizure at school, preceded by fatigue and excessive sleepiness. The seizure lasted approximately 45 minutes, with loss of consciousness, upward eye-rolling, and frothing at the mouth. No urinary or fecal incontinence was reported. The patient had reportedly consumed his usual meals prior to the event, with no history of prolonged fasting or reduced oral intake noted by caregivers.

On ambulance arrival, random blood glucose was 0.9 mmol/L. Intramuscular glucagon was administered, followed by intravenous dextrose (D20) upon hospital arrival. Postictally, the patient was drowsy, hallucinating, and diaphoretic.

One month prior, the patient had refractory supraventricular tachycardia with a heart rate of 240 beats per minute. Vagal maneuvers were ineffective. Adenosine (0.1 mg/kg IV) caused a transient heart rate drop; subsequent doses of 0.2 mg/kg IV ×3 failed. Esmolol infusion (loading dose of 2,000 μg over 1 to 2 minutes, then 25 μg/kg/min) was initiated; increasing to 225 μg/kg/min caused hypotension (blood pressure 60/30 mmHg), which was managed with 25 mL/kg of Ringer’s lactate. Eventually, the patient stabilized with propranolol and amiodarone, which was later discontinued. Follow-up electrocardiogram, Holter, and echocardiography were normal, except for mild mitral regurgitation.

Nadolol 8 mg orally twice daily was started the evening before the seizure. The exact interval between the last nadolol dose and seizure onset could not be precisely determined, but was within 24 hours of initiation. The decision to initiate nadolol was made to provide sustained beta-blockade due to its longer half-life and more stable pharmacokinetic profile compared with propranolol, which requires more frequent dosing [4]. Nadolol is also recognized in pediatric arrhythmia management for its prolonged duration of action and reduced central nervous system penetration [4]. These factors were considered in an attempt to optimize arrhythmia control and improve treatment adherence. However, the longer duration of action may have contributed to prolonged hypoglycemic exposure in this patient. Birth history included an emergency cesarean section for maternal COVID-19 infection. The patient was preterm with a birth weight of 1.5 kg and spent one week in the neonatal intensive care unit. Developmental milestones were normal, and vaccinations were up to date.

Examination revealed an active, well-nourished child with no acute distress. Heart rate was 100 beats per minute, blood pressure 86/50 mmHg, respiratory rate 26/min, oxygen saturation 100%, and random blood glucose 4.5 mmol/L. Chest, cardiovascular, abdominal, and neurological examinations were unremarkable. ECG, chest X-ray, and CT brain were normal.

Investigations showed a C-peptide of 0.18 nmol/L (low), lactic acid 4.2 → 2.7 mmol/L, fasting glucose 4.3 mmol/L, lipid profile with cholesterol 5.9, triglycerides 1.1, high-density lipoprotein cholesterol (HDL) 1.88, and low-density lipoprotein cholesterol (LDL) 3.58, with urine microscopy showing 10-25 red blood cells. Random blood glucose trends ranged from 7.7 to 14.1 mmol/L. Echocardiography showed mild mitral regurgitation with preserved ejection fraction (26-30%). A full critical hypoglycemia panel, including serum insulin, cortisol, ketones, and growth hormone levels, was not obtained at the time of the acute event due to the urgency of seizure management. However, subsequent endocrine evaluation, including C-peptide and fasting glucose assessment, did not suggest intrinsic metabolic or endocrine pathology. A summary of laboratory investigations with corresponding reference ranges is provided in Tables 1-4.

**Table 1:** Glycemic and Endocrine Investigations Reference ranges are institution-specific and age-appropriate. TSH, thyroid-stimulating hormone.

Parameter	Patient Value	Units	Reference Range
Random blood glucose (at seizure)	0.9	mmol/L	3.9-7.8
Random blood glucose (subsequent range)	7.7-14.1	mmol/L	3.9-7.8
Random blood glucose (repeat sample)	10.5	mmol/L	3.9-7.8
Fasting blood glucose	4.3	mmol/L	3.9-5.5
C-peptide	0.18	nmol/L	0.37-1.47
TSH	1.03	µIU/mL	0.67-4.16

**Table 2:** Metabolic and Renal Investigations Reference ranges are institution-specific and age-appropriate.

Parameter	Patient Value	Units	Reference Range
Urea	8.9	mmol/L	1.8-6.4
Creatinine	22	µmol/L	20-44
Sodium	136	mmol/L	136-145
Potassium	4.6	mmol/L	3.5-5.1
Chloride	107	mmol/L	98-107
Bicarbonate	15	mmol/L	20-31
Calcium	2.36	mmol/L	2.20-2.70
Inorganic phosphorus	2.0	mmol/L	1.10-2.20
Magnesium	0.86	mmol/L	0.66-1.07

**Table 3:** Lactate and Cardiac Enzymes Reference ranges are institution-specific and age-appropriate.

Parameter	Patient Value	Units	Reference Range
Blood lactate (initial)	4.2	mmol/L	0.5-2.2
Blood lactate (repeat)	2.7	mmol/L	0.5-2.2
Lactate dehydrogenase	409	U/L	120-300
Creatine kinase	121	U/L	46-171
Troponin I (high sensitivity)	<2.5	ng/L	≤45

**Table 4:** Hematology, Lipid Profile, and Urine Microscopy Reference ranges are institution-specific and age-appropriate. WBC, white blood cell count; Hb, hemoglobin; HDL, high-density lipoprotein cholesterol; LDL, low-density lipoprotein cholesterol; RBC, red blood cells.

Parameter	Patient Value	Units	Reference Range
WBC count	13.84	×10⁹/L	4.4-12.9
Hb	13.0	g/dL	11.4-14.3
Platelet count	280	×10⁹/L	150-400
Total cholesterol	5.9	mmol/L	<5.2
Triglycerides	1.1	mmol/L	<1.7
HDL	1.88	mmol/L	>1.0
LDL	3.58	mmol/L	<3.4
Urine RBC	10-25	Per high-power field	0-5

The patient was admitted under cardiology with continuous cardiac monitoring. IV fluids included ½ normal saline with 5% dextrose at 1.2 L/24 h. Hourly glucose and two-hourly calcium monitoring were performed. Propranolol was titrated to 5 mg every 8 hours post-feeding and held if heart rate fell below 80 beats per minute or systolic blood pressure fell below 90 mmHg. Endocrinology recommended a formal fasting study after discontinuation of dextrose.

## Discussion

Beta-blockers, particularly non-selective agents such as nadolol, can impair hepatic glucose production and blunt adrenergic warning signs. Children under five are especially susceptible due to higher glucose utilization, lower glycogen stores, and limited ability to communicate early hypoglycemic symptoms [1,3].

Although a complete critical hypoglycemia sample was not obtained during the acute event due to emergency stabilization priorities, available clinical and laboratory findings support drug-associated hypoglycemia. The patient had no history of prolonged fasting or reduced oral intake, and fasting glucose levels were within normal limits. There was no strong clinical or laboratory evidence to suggest infection or an underlying metabolic disorder, although comprehensive metabolic screening was not performed. The low C-peptide level supports preserved endogenous insulin regulation and argues against hyperinsulinemic hypoglycemia. Together, these findings support impaired hepatic glucose production as the most likely mechanism, consistent with the known metabolic effects of non-selective beta-blockers.

In this case, the patient had a history of recurrent symptomatic supraventricular tachycardia characterized by episodes of palpitations, marked tachycardia reaching 240 beats per minute, and failure of vagal manoeuvers. Previous management included adenosine administration followed by beta-blocker therapy with propranolol, which initially stabilized the arrhythmia. However, due to concerns regarding recurrence and the need for more sustained beta blockade with less frequent dosing, nadolol was initiated. Shortly after initiation, nadolol was closely followed by a severe hypoglycemic seizure. Low C-peptide levels suggested intact insulin regulation, supporting a drug-induced hypoglycemia rather than intrinsic endocrine dysfunction [1].

Clinical recommendations include regular glucose checks after starting non-selective beta-blockers, especially in children under five, teaching caregivers to recognize early signs of hypoglycemia such as lethargy, diaphoresis, and pallor, and considering safer medication alternatives when appropriate [2,5]. As summarized in Table 5, nadolol differs from propranolol in key pharmacokinetic properties, including a longer half-life, predominantly renal clearance, and a higher risk of drug accumulation, factors that may contribute to prolonged hypoglycemic exposure [4]. Switching from nadolol to propranolol, with careful monitoring, can therefore maintain arrhythmia control while reducing hypoglycemia risk [2,5]. Reporting such adverse events enhances pediatric pharmacovigilance [3].

**Table 5:** Comparison of Nadolol and Propranolol in Pediatric Patients Pharmacologic properties, dosing, and risk profiles relevant to pediatric arrhythmia management. Data adapted from reference [4].

Feature	Nadolol	Propranolol
Beta selectivity	Non-selective	Non-selective
Half-life	14-24 hours	3-6 hours
Metabolism	Minimally hepatic, mainly renal	Hepatic and renal
Accumulation risk	Higher (infants with infrequent stooling)	Lower, shorter half-life
Hypoglycemia risk	Documented, rare but severe	Lower; shorter exposure window
Dose adjustment	Required in renal impairment	Required in hepatic impairment
Clinical pearls	Long-acting; monitor glucose closely	Easier titration; safer in infants; monitor heart rate and blood pressure

## Conclusions

Beta-blocker-induced hypoglycemia is a rare but serious complication in young children. Clinicians should maintain vigilance, monitor glucose, and educate caregivers when initiating non-selective beta-blockers.
